# Phenol Photocatalytic Degradation by Advanced Oxidation Process under Ultraviolet Radiation Using Titanium Dioxide

**DOI:** 10.1155/2013/815310

**Published:** 2013-04-23

**Authors:** Ali Nickheslat, Mohammad Mehdi Amin, Hassan Izanloo, Ali Fatehizadeh, Seyed Mohammad Mousavi

**Affiliations:** ^1^Islamic Azad University, Bandar Abbas Branch, Hormozgan, Bandar Abbas, Iran; ^2^Environment Research Center, Isfahan University of Medical Sciences (IUMS) and Department of Environmental Health Engineering, School of Health, Isfahan University of Medical Sciences (IUMS), Isfahan, Iran; ^3^Research Center for Environmental Pollutants and Department of Environmental Health Engineering, Health Faculty, Qom University of Medical Sciences, Qom, Iran; ^4^Isfahan Water and Wastewater Company, Isfahan, Iran

## Abstract

*Background*. The main objective of this study was to examine the photocatalytic degradation of phenol from laboratory samples and petrochemical industries wastewater under UV radiation by using nanoparticles of titanium dioxide coated on the inner and outer quartz glass tubes. *Method*. The first stage of this study was conducted to stabilize the titanium dioxide nanoparticles in anatase crystal phase, using dip-coating sol-gel method on the inner and outer surfaces of quartz glass tubes. The effect of important parameters including initial phenol concentration, TiO_2_ catalyst dose, duration of UV radiation, pH of solution, and contact time was investigated. *Results*. In the dip-coat lining stage, the produced nanoparticles with anatase crystalline structure have the average particle size of 30 nm and are uniformly distributed over the tube surface. The removal efficiency of phenol was increased with the descending of the solution pH and initial phenol concentration and rising of the contact time. *Conclusion*. Results showed that the light easily passes through four layers of coating (about 105 nm). The highest removal efficiency of phenol with photocatalytic UV/TiO_2_ process was 50% at initial phenol concentration of 30 mg/L, solution pH of 3, and 300 min contact time. The comparison of synthetic solution and petrochemical wastewater showed that at same conditions the phenol removal efficiency was equal.

## 1. Introduction

The most important problem that can threaten the water ecology and public health is the toxic and resistant compounds that can release to the environment through industrial wastewater [1]. Among the chemical compounds that are present in industrial wastewaters, the phenol and its derivatives are prevalent in industrial effluent; in addition, they can be released to water resources through natural ways (degradation of algae or of organic vegetation). Also, due to the physical structure, phenol was found in chemical solutions and even in the municipal wastewater. Due to relatively stability in environment, solubility in water, high toxicity, and associated health problems, phenol removal from industrial wastewater is important [2].

The phenol compounds can be released to environment through some industrial wastewater including coal industry, resin industries, paint industries, pesticides, medicine and cosmetic products, oil refinery, petrochemical, coal mines, aluminum and steel industries, compost, car production, and chemical industry, which can lead to water resources contamination. Phenol is also found in cleaning materials and disinfectants, cigarettes, car exhausts, and some pesticides [3, 4].

Phenol is involved in toxic pollutants, and the US Environmental Protection Agency reported that phenol is a priority among pollutants group [5]. Due to human health effect of phenol, the restrict standards was passed. World Health Organization (WHO) recommended that phenol concentration in water resources entering conventional water treatment must be <2 *μ*g/L. Furthermore, the concentration of phenol, chlorophenols, 2, 4, 6 trichlorophenol in drinking water must be <0.1 *μ*g/L [6]. According to USEPA standard, and the permitted levels of phenol in water resources for human use and for fisheries are 0.3 and 2.6 mg/L, respectively. Furthermore, based on the standards of Japan, the permitted level in industrial wastewater effluent is 5 mg/L [7].

Some methods were used in the treatment of wastewaters containing resistant organic matters such as phenol. Degradation of phenol usually takes place by physicochemical methods including adsorption using activated carbon [8, 9], biological treatment [10], emulsion liquid membrane [11], ion exchanges, advanced oxidation processes (AOPs) (including cavitation, Fenton, and photocatalyst) [10, 12], chemical oxidation (methods in which ozone and water are used), photochemical and electrochemical, and ultrasound waves such as sonochemical, photochemical, photosonochemical [13], and hybrid of mentioned methods.

The hydrothermal oxidation process consists of wet air oxidation and oxidation in subcritical, critical, and supercritical water and was used for phenol degradation, but due to their high cost and energy consuming, they are used in special conditions.

Biological degradation usually takes longer time and is often affected by temperature variations and phenomenon of toxic pollutants [2, 14]. The ion exchanging and adsorption processes are very expensive [2]. However, advanced oxidation processes (AOPs) are among the most effective processes in degradation of resistant compounds.

Generally, AOPs include processes in which active hydroxyl radicals (OH^∙^) as a strong oxidant for degradation and destruction of polluting materials are produced using different methods. Due to high oxidation capacity of hydroxyl radicals (2.8 V), most of the AOPs are based on this active radical [15, 16]. One of the most effective methods of advanced oxidation is the use of UV ray and oxidant such as H_2_O_2_, O_3_, and TiO_2_ [4, 17]. In recent years, common processes were used in the removal of organic matter including Fenton, photofenton, UV/TiO_2_, and UV/H_2_O_2_/O_3_. In photocatalytic degradation, the pollutants are degraded under the UV radiation in the presence of particles of metallic oxides such as ZnO and TiO_2_ [8, 14, 18]. Titanium dioxide is a metallic oxide well known for degradation of organic matters [19] and is of relatively low cost, nontoxic, and insoluble in water [20, 21].

UV/TiO_2_ process is one of the latest and most effective methods for the treatment of these pollutants. In this process, titanium dioxide nanoparticles were extensively used in photocatalytic reactions as a catalyst. The small particle size of this metallic oxide can lead to the increase of special surface of catalyst and the improvement of the photocatalytic activity. In recent research, titanium dioxide nanoparticles in anatase crystalline phase are used and in order to description of produced, XRD spectrum and their SEM images was used [22, 23].

When titanium dioxide was used as suspension form, nanoparticles separation from liquid needed in which separation needed to high investment and operational costs. Also, in this method, thedesigning and operation at continuous system were impossible. Thus, stabilizing titanium dioxide on a solid film caused the removal of the above difficulties and made the industrial application of these processes possible. The improvement of AOP and its application in the industrial projects necessitate the stabilization of photocatalyst on a solid film.

Various methods were applied for the synthesis of titanium dioxide photocatalyst. These methods are divided into two main groups: (1) wet chemistry methods such as sol-gel method and (2) dry method such as aerosol [24, 25]. Also, different methods are used for the preparation of titanium dioxide films such as chemical vapor deposition, hot oxidation, electron beam evaporation, sol-gel method with rotating coating, and dip coating of titanium dioxide compound [26, 27].

In recent year, sol-gel technique is a new method that is used for the synthesis of mineral oxide materials in low temperatures and in nanoscale. In fact, sol-gel is an efficient physical/chemical method for producing materials in form of powder, ceramic coating, fibers, thin layers, and porous materials. In this method, hydrolysis and repetitive condensation reactions of an alkoxide in presence of humidity can produce mineral oxides with particle size that is adjustable from nanometer to the micron:

(1) $$\begin{array}{c} \text{Precursors}\overset{\text{Hydrolysis}}{\to }\text{Sol}\overset{\text{Condensation}}{\to }\text{Gel}. \end{array}$$

**Figure 1:**
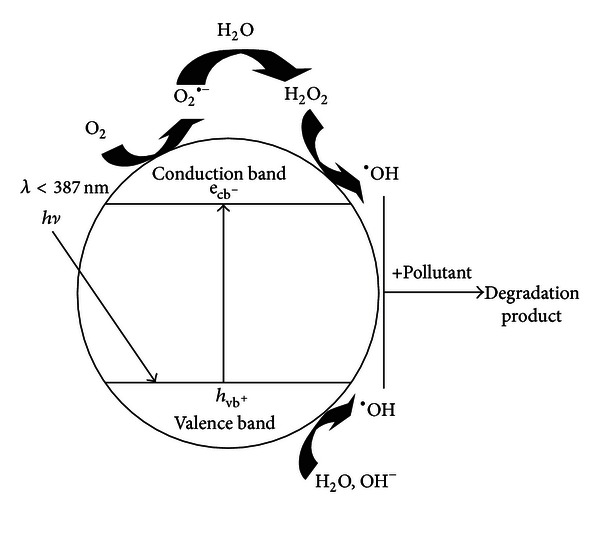
Schematic of photocatalytic mechanism of TiO_2_.

In photocatalytic degradation, light energy was used in photon form with wavelength less than 387.5 nm as ultraviolet radiation or sunlight. The exposure of electrons surface of titanium atoms with light was caused exciting the surface electron and moving from valance layer to transition layer (e_CB_-). The changing in energy level can form the free electron in form of OH^∙^ or other radicals that they can oxidize organic matters and reduced of metals [2, 8, 14, 28]. Photocatalytic destruction of phenol follows the first-order reaction (Figure 1). 

The advantages of AOP were including the low cost, stability, and high efficiency [16, 28].

This study was attempts to coating layers of titanium dioxide on the inner and outer surface of quartz tubes and application of AOP (UV/TiO_2_) for phenol remove from industrial and petrochemical wastewater.

## 2. Materials and Methods

### 2.1. Fixation of TiO_2_ on Surfaces of Quartz Tubes

**Figure 2:**
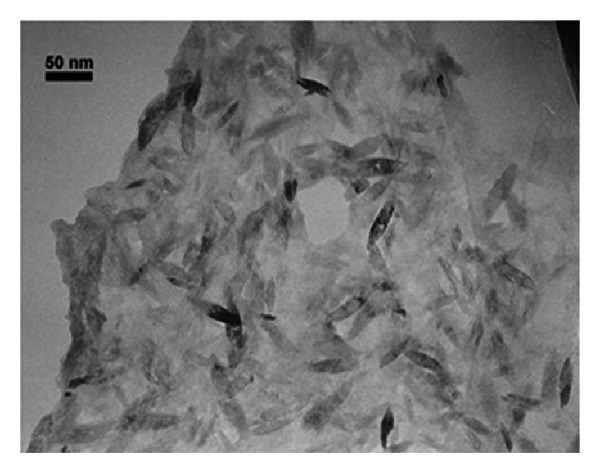
TEM image of titanium dioxide nanoparticles.

**Figure 3:**
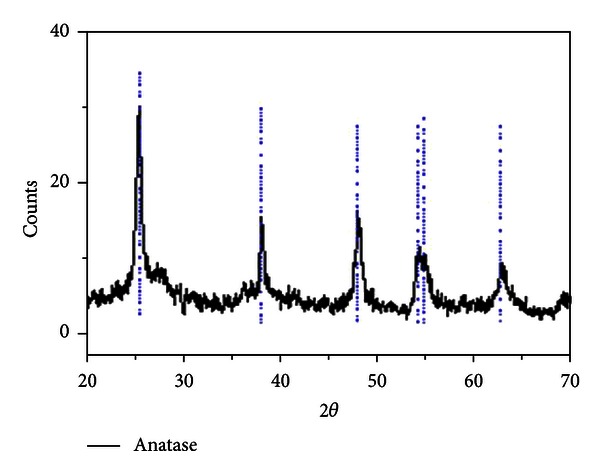
XRD spectrum related to anatase TiO_2_ nanoparticles.

For production of photocatalytic coatings, a solution from nanoparticles of TiO_2_ with 1% (w/w) was prepared. The TiO_2_ nanoparticles were purchased from Nanosav Co. with specification of SAV2104. The TiO_2_ has an oval shape with diameter of >30 nm, and the anatase crystalline phase is formed in water. The TEM image of TiO_2_ nanoparticles and XRD spectrum of anatase TiO_2_ nanoparticles are shown in Figures 2 and 3. 

### 2.2. Preparation of Underlayer

For proper coating of nano layers on surface, there should be no presence of any contamination on the underlayer as it can affect the quality of the coats and create nonuniform layers. Underlayers are comprised of quartz tubes with 20 cm lengths. Cleaning method of underlayers is follows as.

1. Washing up with soap and water to remove the contaminants and fats.
2. Acetone washing.
3. Heating up in a kiln to 500°C for 30 min.
4. Placing underlayers in H_2_O_2_ : H_2_SO_4_ solution at 3 : 7 ratio (w/w) for 1 h. For this purpose, H_2_O_2_ was slowly added to sulfuric acid to prevent bubbles formation. This solution was called piranha solution (strong oxidant) and was able to produce hydroxyl groups on the surface and create them in a hydrophilic way.
5. After washing underlayers with deionized water, were remained in NH_4_OH : H_2_O_2_ : H_2_O solution (1 : 1 : 5 w/w) for 1 h washed, with deionized water, and dried. After this, glass underlayers are ready for coating.

### 2.3. Coating

**Figure 4:**
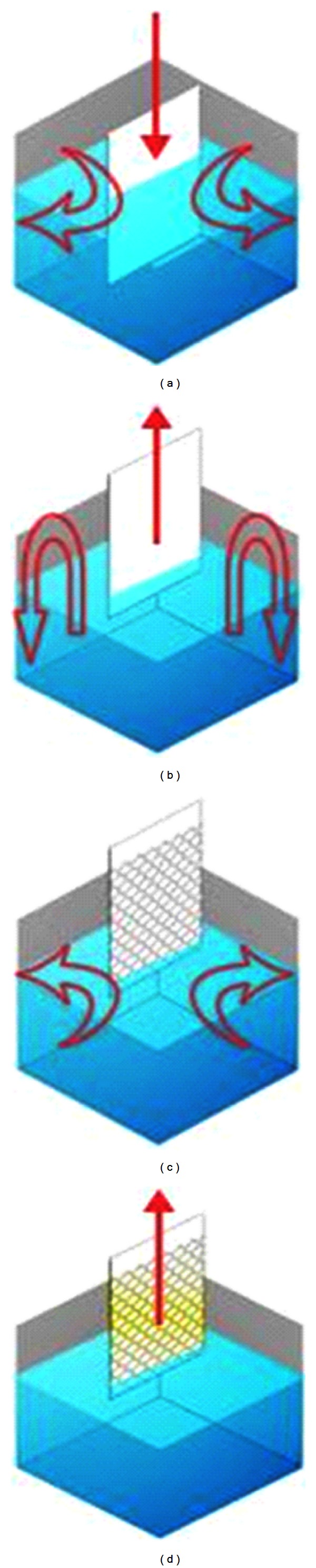
Stages of dip-coating method.

**Figure 5:**
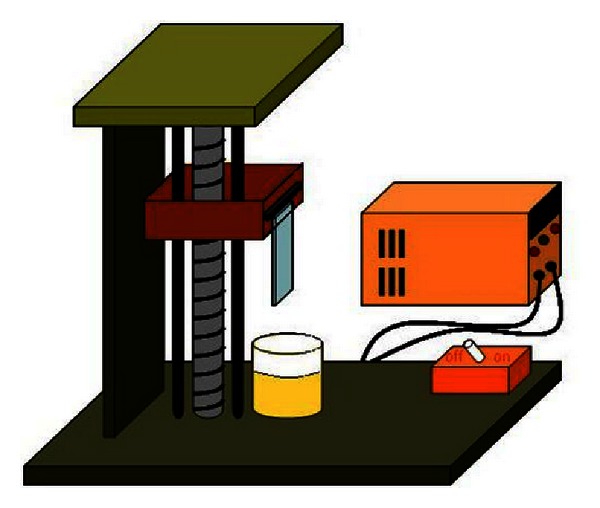
The schematic of dip-coating set up.

**Figure 6:**
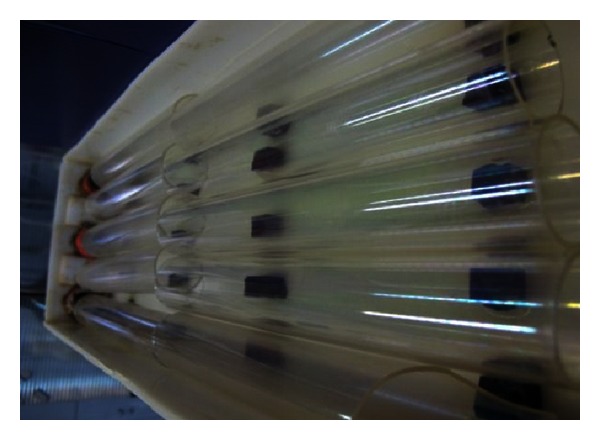
The quartz tubes coated with TiO_2_ nanoparticle.

The used coating method was a dip coating. In this method, coat layers are formed by dipping the under layer into solution and extracting them at constant speed. Thickness of coat was determined by speed of extracting and solution viscosity. Dip coating is an efficient and quick method, but it was suffering from some disadvantages including difference between thickness-coated layers. The thickness of top layers is slightly less than bottom layer.  Figure 4 shows the system used for coating. The cleaned glass under layer was connected to mechanical hoist, and under layer was vertically dipped into nanoparticle cell and kept for 1 min till the solution becomes undistributed. So, the under layer was extracted at a constant speed, and layer thickness was controlled by variation of speed of hoisting up. Figures 4, 5, and 6 showed stages of dip coating, schematic of dip-coating set up method, and quartz tubes coated with TiO_2_ nanoparticle, respectively. To perform the coating of quartz tubes dip-coating method, 180 mL of TiO_2_ nanoparticle solution was used to create coating layer with thickness *≈*400 nm on the surfaces of tubes.

### 2.4. Photocatalysis Reactor Set Up

**Figure 7:**
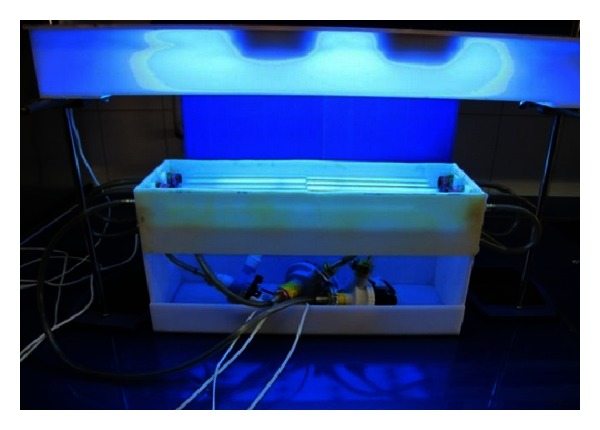
Photocatalysis laboratory-scale plant.

The photocatalysis reactor consisted of 8 coated quartz tubes with TiO_2_ nanoparticles with 30 mm inside diameter as parallel arrangement and contact tank. The contact tank was constructed of plexiglass with dimensions of 50 : 10 : 15 cm and 3.5 L working volume. The quartz tubs were immersed in open top water bath. The feed solution was introduced to system by means of two peristaltic pumps and flowed inside/outside tubes at uniform constant speed. In photocatalysis reactor, in order to provide UV radiation, four UV lamps with maximum wavelengths of 365 nm and 250 w were used on top of reactor. The schematic of photocatalysis reactor and applied UV lamps is shown if Figure 7.

### 2.5. Operational Conditions

An investigation program comprising 5 different phases was performed. Each phase corresponded to certain intensity of light and distance from UV source (15, 30, 50, 100, and 150 cm), initial phenol concentrations (30, 40, 60, 80, and 100 mg/L), solution pH (3, 6.5, 9, and 11) contact time (from 0 to 300 min), and phenol removal of Bandar Abbas oil refinery wastewater. All experiments were performed at 25°C.

### 2.6. Chemicals and Instrument

All chemical substances were purchased from Merck Co. The amounts of phenol were analyzed using the standard methods [29]. The spectrophotometer was used for phenol analysis at wavelength of 670 mm.

## 3. Results

### 3.1. Optical Properties of TiO_2_ Layers

After coating glass underlayers, their optical transmission spectrum was determined by spectrophotometer UV-Vis at wavelength range of 200 to 1100 nm accuracy of 1 nm and scanning speed of 400 nm/min and air as reference.

**Figure 8:**
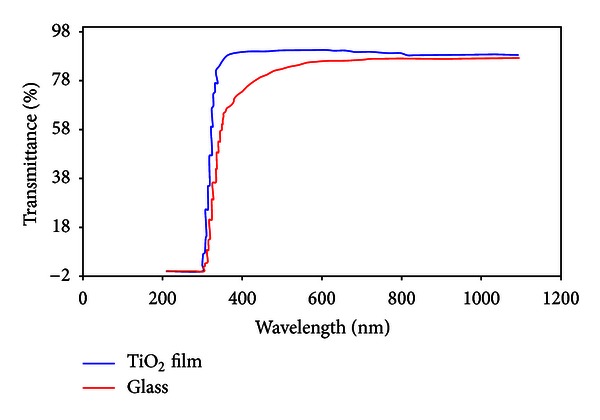
Optical transmission spectrum through glass only and a thin TiO_2_ layer (coating speed: 3 mm/sec).

The TiO_2_ (1% wt) cell was used for coating with 3 mm/sec for speed of coating layers. As shown in Figure 8, after first layer coating, the edge of TiO_2_ film adsorption has slightly moved from glass position which indicated that coating process successfully performed. This slight movement showed that TiO_2_ film deposit on glass was low and thin. TiO_2_ is a semiconductor with indirect energy in range of 3–3.2 ev, and it could not properly absorb light in the range of UVA (320–380 nm) which includes sun UV rays. For this reason, edge of adsorption could not clearly appear at thin film of TiO_2_, and oscillations could not be observe due to interference effect. Therefore, only a thin film of TiO_2_ was produced.

**Figure 9:**
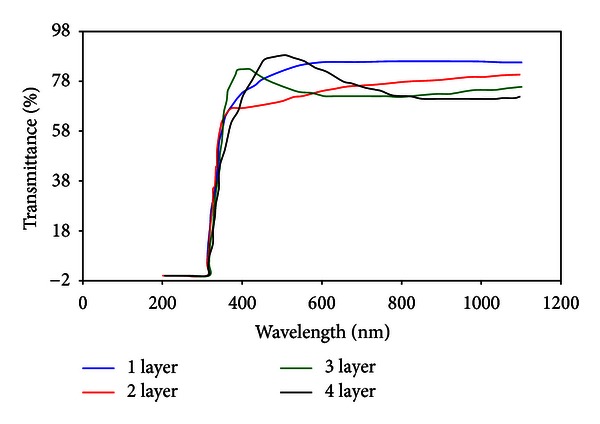
Optical transmission spectrum through some layers of TiO_2_ after each coating.

To increase layers thickness, coating was repeated few times. Optical transmission spectrum of TiO_2_ film after coating layers was depicted in Figure 9. For preventing the removal of TiO_2_ film of tube surface and for confident estimating of TiO_2_ film thickness, after each coating, layers were placed in a kiln at 200°C for 10 min. In this way, the layers were made dry and hard.

According to Figure 9, at first coating, the transmittance of visible light was 85%, and the transmittance of visible light was reduced with the increasing number of coat layers (thickness of TiO_2_ film). This reduction in transmittance may be related to interference effects and light scattering. In this stage, the coating speed was 3 mm/sec, and after each layer, layers were placed in a kiln at 200°C for 10 min.

**Figure 10:**
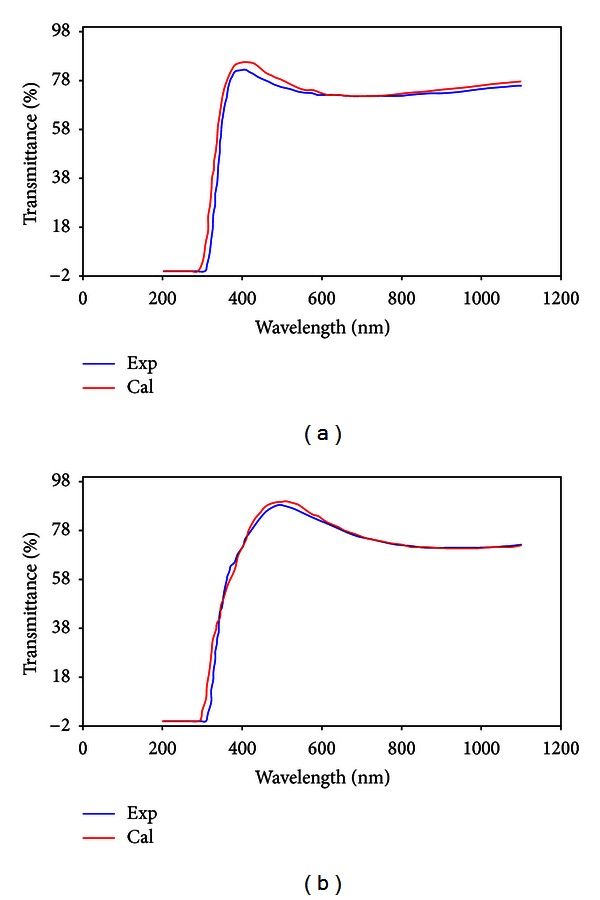
Experimental and calculated optical transmission spectrum of TiO_2_ layers: (a) third layer and (b) fourth layer.

In a thicknesses less than 100 nm, optical transmission spectrum was not indicating oscillations due to interference effects. For this, the optical transmission spectrum could not be done for the first and second layer. As the thickness of TiO_2_ film was increased, the effect of oscillations clearly appeared.  Figure 10 showed the results of optical transmittance for the third and fourth layers of TiO_2_ film.

As seen in Figure 10, the experimental and calculated transmitted spectrum in wavelengths of 400–1100 nm was agreed to large extent. Results showed that in monolayer coating, 30 nm of TiO_2_ film was adsorbed on under layer, and after dozen coating, the TiO_2_ film thickness reached to 360 nm.

### 3.2. Effects of Intensity of Light and Distance from UV Source

**Figure 11:**
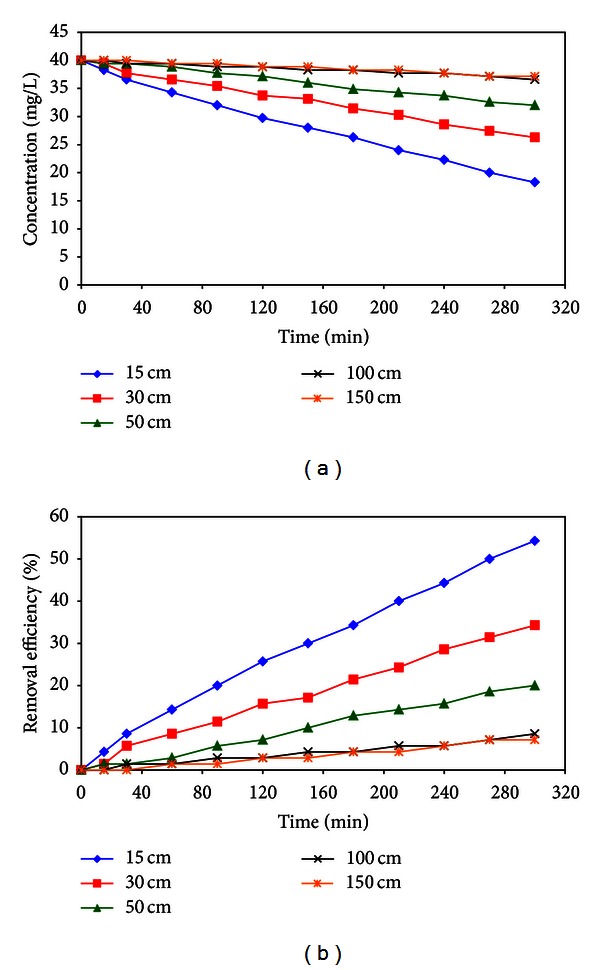
Effect of various intensities of UV lamps on (a) effluent phenol concentration and (b) removal efficiency of phenol (*C*_0_: 40 mg/L, solution pH: 6.5, and contact time: 0–300 min).

Distance of UV lamps from phenol solution and water was adjusted to 15, 30, 50, 100, and 150 cm. The results of the effects of intensity of UV source are shown in Figure 11. The phenol removal by photocatalysis was straight line decreased with the rising of UV lamps from reactor.

As in Figure 11, it appeared that optimum distance in which concentration reduction occurs most rapidly was 15 cm from surface of phenol solution. The reason for this may be due to lower light intensity and reduction of surface photocatalytic activities.

### 3.3. Effect of Initial Phenol Concentrations and Contact Time

**Figure 12:**
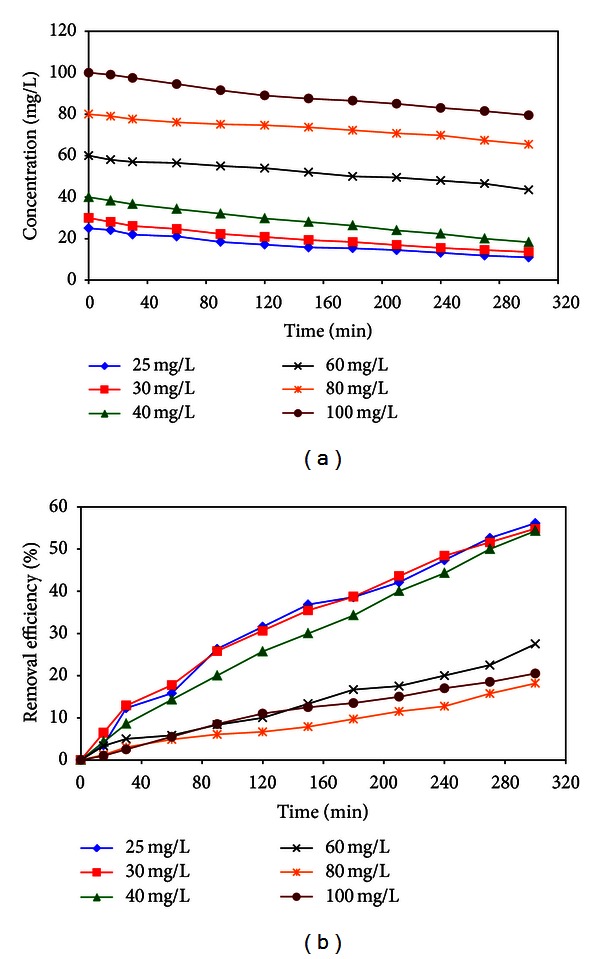
Influence of initial phenol concentration and contact time (a) effluent phenol concentration and (b) phenol removal efficiency (*C*_0_: 25–100 mg/L, solution pH: 6.5, distance of UV source: 15 cm, and contact time 0–300 min).

To assess the effect of initial phenol concentrations, the solutions with concentrations of 30, 40, 60, 80, and 100 mg/L were provided and were fed to reactor.  Figure 12 showed the results related to of effect of initial phenol concentrations on photocatalysis phenol removal efficiency. In terms of Figure 12, the phenol removal at initial concentration of 25, 30, and 40 mg/L by photocatalysis laboratory-scale plant was about 50% at 300 min contact time.

The percentage of phenol removal (Figure 12) decreases with the mounting of the initial concentration of phenol from 50% to 20%. This could be due to the saturation of the surface coat of photocatalyst with by-product that resulted from degradation of phenol. As clearly seen from Figure 12, the increase in the duration of photocatalysis process for over 5 h led to further diminishment in phenol concentration.

### 3.4. Effect of pH

**Figure 13:**
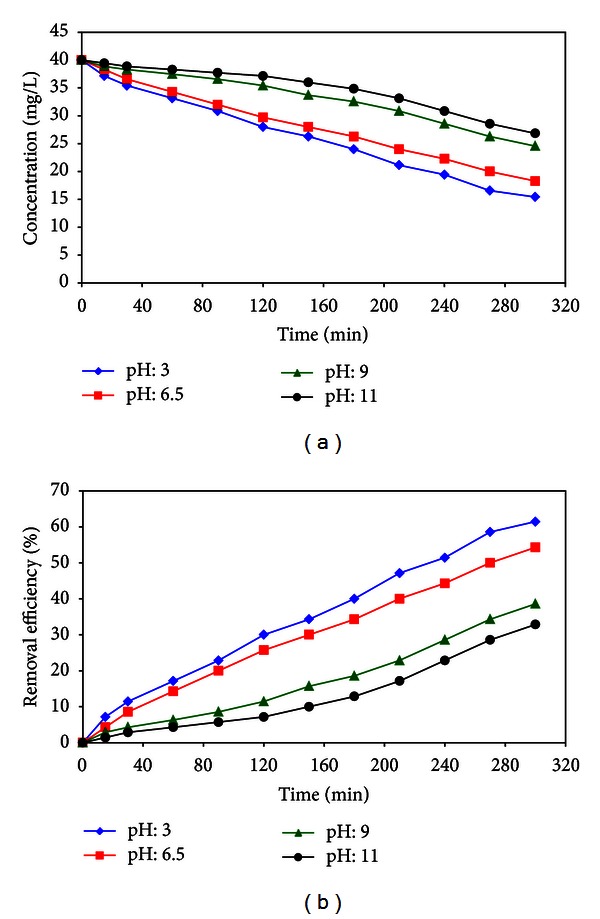
Effect of solution pH on (a) effluent phenol concentration and (b) phenol reduction (*C*_0_: 40 mg/L, distance of UV source: 15, contact time 0–300 min, and various solution pH).

The reduction of phenol was studied from the aqueous solution at different pH values. The solution with phenol concentration of 45 mg/L was provided, and HCl and NH_4_OH were used in order to adjust solution pH at values of 3, 6.5, 9, and 11. The results obtained are shown in Figure 13 which shows the effect of solution pH on the phenol removal from the aqueous solution by photocatalysis process expressed in terms of the effluent phenol concentration and phenol removed percent. It is clear that phenol was effectively removed at solution pH: 3.

### 3.5. Phenol Removal from Bandar Abbas Oil Refinery Wastewater

**Figure 14:**
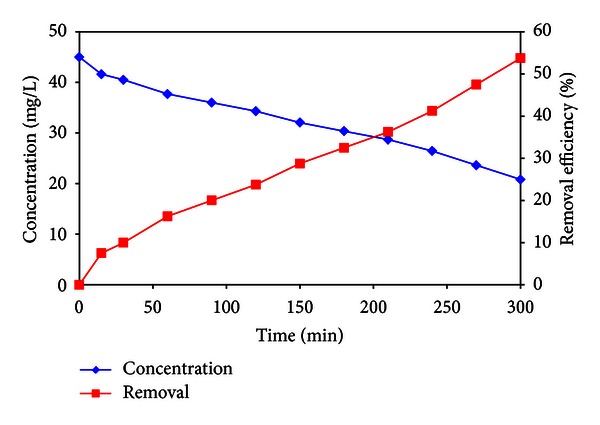
Phenol removal from Bandar Abbas oil refinery wastewater with phenol concentration of 45 mg/L.

This study was performed in which a pilot scale photocatalysis system was fed from Bandar Abbas oil refinery wastewater. The composition of the Bandar Abbas oil refinery wastewater varied as follows: phenol concentration (45 mg/L) and pH (5.5).  Figure 14 shows the evolution of phenol removal from Bandar Abbas oil refinery wastewater.

## 4. Discussion

Photocatalytic degradation of phenol with TiO_2_ as catalyst and UV radiation is the newest process. In this research, photocatalytic degradation of phenol with emphasis on contact time, solution pH variations, UV radiation, and initial phenol concentration was assessed.

### 4.1. TiO_2_ Coating Layers

In the first stage of this research, solution of the cell containing TiO_2_ nanoparticles with anatase crystalline was prepared. So, coating of solution of cell containing crystallized nanoparticles inside/outside quartz tubes using dip-coating (sol-gel) method was induced. The particles size, crystalline structure of coated surface with X-ray tests, and SEM were assessed. Results indicated that the obtained nanoparticles had anatase crystalline structure with average particles size of 30 nm and were uniformly distributed over tube surface. Also, results showed that in monolayer coating, 30 nm of TiO_2_ film was adsorbed on under layer, and after dozen coating, the TiO_2_ film thickness reached to 360 nm. It was clear that light could easily pass through the four layers of coating (1.5 mm).

### 4.2. Photocatalytic Degradation of Phenol

These results indicated that increasing contact time and reducing initial phenol concentration led to improvement in the removal percentage of phenol. Results of the experiment showed that in this process, removal efficiency was higher in low solution pH. So, with rising the solution pH from 3 to 11, phenol removal efficiency varied from 60% to 30%.

As mentioned previously, from assessment of initial phenol concentration and with regard to contact time and solution pH, it could be deducted that for lower concentration of phenol in wastewater, removal efficiency is higher. The effect of solution pH showed that pH is an important variable and plays an important role in the equilibrium of acid and alkalinity, thereby, affected on degraded and nondegraded concentration of phenol. The maximum phenol efficiency was related to solution pH of 3 at phenol concentration of 30 mg/L. At constant amount of TiO_2_ film on tubes, increasing initial phenol concentration resulted in low phenol removal efficiency and greater contact time needed. It can be predicted that by the increasing TiO_2_ contact surface, the removal of higher concentrations will be possible. Comparison obtained results of phenol degradation from laboratory sample and industrial wastewater showed that at similar conditions, the phenol removal was equal.

Photocatalysis laboratory scale studies showed that this process could be suggested as an applicable method for removing phenol as toxic and dangerous matter from effluent and wastewater by degradation and converting it into safe matter. Application of these studies on laboratory scale could provide proper information for using this process as full scale.

It can be expected that AOP including UV/TiO_2_ with advantages such as nonproduction of by-products, production of nondangerous by-products, environment friendly, and high efficiency in comparison to the biological methods will have a suitable layout in water and wastewater industries. Photocatalytic UV/TiO_2_ process in conjunction with other processes such as membrane process, application of fixed TiO_2_ film (present research), and changing of semiconductor TiO_2_ surfaces and replacing of visible light in lieu UV radiation could be one of the most effective methods for the removal of organic solutions including phenol and its compounds. In addition, we suggested that other studies should be performed to test this method for some resistance and toxic compounds.
